# From open to robotic distal pancreatectomy: the learning curve and mastering benchmark outcomes in a Scandinavian high volume center

**DOI:** 10.1007/s00423-025-03855-w

**Published:** 2025-10-10

**Authors:** Paul S. Krohn, Kristian S. Kiim, Christoph Tschuor, Daisuke Fukumori, Stefan K. Burgdorf

**Affiliations:** Department of Surgery and Transplantation, Rigshospitalet University Hospital, Inge Lehmanns Vej 7, Copenhagen, 2100 Denmark

**Keywords:** Pancreas, Adenocarcinoma, Robot, Technique, Survival

## Abstract

**Background:**

Robotic distal pancreatectomy is increasingly utilized for the treatment of both benign and malignant lesions in the body and tail of pancreas. The aim of the current study was to report outcomes of the implementation of robotic distal pancreatectomy in a high-volume center with special emphasis on the learning curve.

**Methods:**

This was a retrospective single-center study of consecutive patients undergoing robotic distal pancreatectomy performed by a dedicated team of three surgeons at a high-volume HPB center from September 2019 to November 2023. Patients with borderline or locally advanced tumors, or ingrowth in neighboring organs were not included for robotic approach.

Intra- and postoperative outcomes were registered and compared across three pre-defined time periods of the first 40, middle 40 and remaining 40 procedures. Cumulative sum analysis was performed for the outcomes duration of surgery and textbook outcome.

**Results:**

A total of 120 consecutive patients were included. The mean duration of surgery decreased significantly from 264 min to 239 min and lastly 222 min per procedure (*P* = 0.003) and the learning curve for this outcome was reached after 70 cases. The rate of splenic preservation was highest in the last period (12.5% vs. 12.5% vs. 30.0%, *P* = 0.066). The rate of textbook outcome across the three time periods increased (62.5% vs. 70.0% vs. 82.5%) and most benchmark values were obtained in the third period. The learning curve effect reached benchmark outcome values after 60 procedures and achieved better than expected outcomes after 90 procedures.

**Conclusions:**

Robotic distal pancreatectomy was safely implemented in a high volume HPB-center going from open to robotic approach. The learning curve was surpassed after 19 procedures reaching benchmark outcome values after 60 procedures.

## Introduction

Distal pancreatectomy (DP) is the treatment of choice for most resectable lesions of the body and tail of the pancreas [1]. Although first described in 2003, the robotic approach for distal pancreatectomy has only recently become routine in many centers [2–5]. With new evidence emerging at a rapid pace, several studies, including level 1 evidence, have reported equal or reduced blood loss, rates of operative intervention and postoperative pancreatic fistulas as well as time to hospital discharge [6–9]. As with all new techniques, a learning curve needs to be surpassed with robotic DP (RDP) [10]. A recently published large multicenter study on learning curve in minimally invasive DP pooled laparoscopic and robotic procedures due to a paucity of cohort studies of relevant size [11]. For robotic pancreatic surgery to evolve, literature describing the outcomes of this approach is vital. A recent multicenter study described the learning curves for RDP, emphasizing duration of surgery as a measure of competency, severe complications as proficiency and textbook outcome as mastery, reaching these plateaus at 46, 63 and 73 procedures, respectively, and further evaluated in a systematic review [12, 13].

The aim of the current study was to report the outcomes of implementation of RDP in a single high-volume center shifting from the open to robotic approach, with an emphasis on examining the number of procedures needed to surpass the above-mentioned learning curves in a dedicated team.

## Materials and methods

This was a retrospective single-center study on the first 120 consecutive patients undergoing elective RDP at Rigshospitalet, Copenhagen university hospital in Denmark. This is a tertiary referral center for 2.3 million people. The annual number of pancreatic resections in our center is steady around 200. The study period was from September 2019 to November 2023 and data were gathered prospectively in a local database.

### Training and patient selection

Prior to the implementation of a robotic approach all surgeons scheduled to perform robotic DP received formal robotic training. The training program consisted of 40 h of robotic platform simulation training per surgeon followed by dry laboratory training and subsequently wet laboratory training on pig models. Prior to the introduction of the robotic program all surgeons had extensive experience with open pancreatic surgery, however no experience with laparoscopic pancreatic surgery.

Patients were all suspected of malignant or premalignant disease in the body or tail of the pancreas and evaluated as being resectable at a multidisciplinary team conference on the basis of a contrast enhanced triphasic thoracoabdominal CT.

In the implementation phase there was a selection of patients suited for the robotic approach, i.e. localized smaller tumors not involving neighbor organs. However during the study period only patients with borderline resectable or locally advanced tumors according to Isaji et al. [14] or with contraindications to pneumoperitoneum or minimally invasive surgery underwent open repair. There were no specific criteria in regard to previous upper GI surgery or tumor size, however thorough selection of less complicated cases was done in the beginning of the study period. Thus, as confidence and experience grew, the complexity of patients selected for robotic approach increased. The overall aim was to use the same operative strategy as performed in open surgery using central vascular control and RAMPS technique, thus not changing the operative strategy, only the approach. Before the implementation of robotic DP, the preferred approach was open and there was no prior experience with laparoscopic pancreatic surgery.

### Surgical technique

After general anesthesia, a gastric tube and a urinary catheter was placed. The robotic procedure was performed with the DaVinci Si or Xi surgical robot (Intuitive Surgical, California, USA) placing a total of four robotic trocars, one 15 mm as well as an 8 mm Airseal^®^ trocar for the DaVinci Xi and three robotic trocars, one 12 mm, one 15 mm and an 8 mm Airseal trocar for the Da Vinci Si. After insufflation the patient was placed in a reverse Trendelenburg position and the robot was docked. Intraoperative ultrasonography of the liver was performed to rule out metastases. After entering the lesser sac by division of the gastrocolic ligament. The pancreas was then mobilized from the retroperitoneum and divided using a stapler (Signia™, black re-inforced cartridge, Medtronic, Minneapolis, USA) with preservation of the splenic vessels. In case of suspected malignancy in the pancreas a splenectomy was also performed according to the RAMPS technique, thus necessitating the division of the splenic artery and vein. As a standard, splenic preservation was attempted whenever malignancy was not suspected, and this did not change during the study period. The organ(s) was removed from the abdomen in an endobag using a small midline or Pfannenstiel incision. A drain was placed at the divided pancreas body, and no remnant coverage of the divided pancreas was done.

### Perioperative management

Postoperatively patients followed a standard enhanced recovery after surgery program and were mobilized on the day of surgery and allowed early oral feeding with no restrictions. In case of splenic preservation only perioperative antibiotic prophylaxis was administered, patients undergoing splenectomy received antibiotics the first three postoperative days. The drain remained in situ until postoperative day four, with standard drain fluid amylase examination on postoperative day three and four.

Patients were discharged from the hospital when they were sufficiently pain relieved on oral analgetic treatment, had sufficient oral intake and otherwise able to perform sufficient self-care. All patients were seen in the outpatient clinic after two weeks or by indication.

### Study variables and definitions

Preoperative variables registered included patient sex, age at time of surgery, body mass index (BMI), American Society of Anesthesiologist’s (ASA) score and smoking status. Intraoperative variables were duration of surgery, splenic preservation, intraoperative complications, need for additional resection due to frozen section analysis, conversion to open surgery and blood loss (retrieved from anesthesia records). The duration of surgery included potential time waiting for frozen section results. Postoperative variables included complications graded according to the Clavien-Dindo score, time to hospital discharge, unplanned readmission and 30-day mortality. For patients undergoing RDP due to adenocarcinoma the lymph node yield, resection margin, and adjuvant treatment was also reported. Resection margins were defined as R0 (> 1.5 mm to the tumor), R1 (< 1.5 mm to the tumor) and R2 (macroscopic resection in tumor) [15]. If an R1 resection was due to intimate relation to the anterior or posterior surface this was noted.

To further evaluate the outcomes all patients were graded according to the Dutch Pancreatic Cancer Group Textbook outcome, which is a composite score reflecting that none of the following events occurred: Clavien-Dindo complication ≥ 3, postoperative pancreatic fistula, postoperative hemorrhage, readmission or 30-day mortality [16].

### Statistical analysis

Continuous variables were reported as mean (standard deviation, SD) or median (range) where appropriate and compared across study periods using Student’s t-test. Categorical variables were reported as n (%) and compared using the Chi-squared test. To evaluate the potential progress in learning curve the study cohort was divided into three different groups based on the number of patients subjected to RDP. The first time period included the initial 40 patients, hereafter the subsequent 40 patients and the third time period the remaining 40 patients. The duration of surgery was depicted using a scatter plot including a regression line and the survival of patients with pancreatic ductal adenocarcinoma (PDAC) was calculated using the Kaplan-Meier method and illustrated using a Kaplan-Meier plot.

A cumulative sum (CUSUM) analysis was applied to examine the learning curve of the outcomes duration of surgery and textbook outcome. The CUSUM approach has been described in detail elsewhere [17]. Briefly, analysis of the CUSUM plots of continuous outcomes indicate when performance is transforming from the initial (leaning) phase to proficiency and subsequent mastery. For binary outcomes, CUSUM plot analysis can identify if an outcome is stable and acceptable, better than expected or spiraling out of control. For the analysis of textbook outcome, the accepted failure probability was set to 30% and smallest detectable failure probability to 10%.

P-values < 0.05 were considered statistically significant. Data were analyzed using R software version 4.0.2 (R Foundation for Statistical Computing, Vienna, Austria). This study was written according to the STROBE guidelines [18]. The study was approved by the by the local hospital board as part of a quality assurance project.

## Results

During the study period a total of 120 patients underwent RDP at our institution, and in the same period 76 open DP were performed. The mean age of patients was 63.6 (range 22–86) years and 55 (45.8%) were females. The most common indication for RDP was PDAC (27.5%) followed by intraductal papillary mucinous neoplasm (IPMN, 26.7%) and neuroendocrine tumor (21.7%). A total of 58 (48.3%) patients had no complications postoperatively and the median length of stay was 6 (range 4–52) days. In a total of 22 (18.3%) of the procedures the spleen was preserved, and the mean blood loss was 203.5 ml. The mean duration of surgery was 242 (SD 65) minutes and significantly less in patients where splenic preservation was performed (212 vs. 248 min, *P* = 0.013). No patients required intraoperative blood transfusion.

Nine (7.5%) procedures were converted to open surgery. Reasons for conversion was subcutaneous emphysema (*n* = 2), positive resection margin at intraoperative frozen section examination requiring total pancreaticoduodenectomy (*n* = 2), adhesions (*n* = 1) and technical difficult procedure (*n* = 4). Postoperatively a total of three patients underwent laparotomy due to postoperative hemorrhage (*n* = 2) and perforated stomach (*n* = 1).

Textbook outcome was obtained for 86 (71.7%) of the patients. Data for the entire cohort is given in Table 1.

**Table 1:** Pre- intra- and postoperative data on patients undergoing robotic distal pancreatectomy

Variable	Level	Total (*n* = 120)
Age [years]	mean (sd)	63.6 (13.8)
Sex	Female	55 (45.8)
	Male	65 (54.2)
BMI [kg/m^2^]	mean (sd)	26.6 (4.7)
ASA score	I	7 (5.8)
	II	63 (52.5)
	III	50 (41.7)
Additional resection		5 (4.7)
Splenic preservation		22 (18.3)
Intraoperative blood loss [ml]	mean (sd)	203.5 (334.8)
Conversion to open surgery		9 (7.5)
Duration of surgery [minutes]	mean (sd)	241.6 (65)
Length of stay [days]	median [range]	6 [4, 52]
Postoperative pancreatic fistula	None	73 (60.8)
	Biochemical	27 (22.5)
	Grade B	20 (16.7)
Clavien-Dindo complication grade	0	58 (48.3)
	1	17 (14.2)
	2	23 (19.2)
	3a	15 (12.5)
	3b	5 (4.2)
	4	2 (1.7)
Textbook outcome		86 (71.7)
Pathology	Adenocarcinoma	33 (27.5)
	IPMN	32 (26.7)
	Neuroendocrine	26 (21.7)
	Other	29 (24.2)
Lymph nodes	median [range]	13 [0–65]
Readmission		17 (14.2)
Death during follow-up		9 (7.5)
Follow-up [months]	median [range]	23.2 [3.2–52.9]

BMI: Body mass index. ASA: American Society of Anesthesiologists. ISGPS: International Study Group of Pancreatic Surgery

### Time periods

**Fig. 1:**
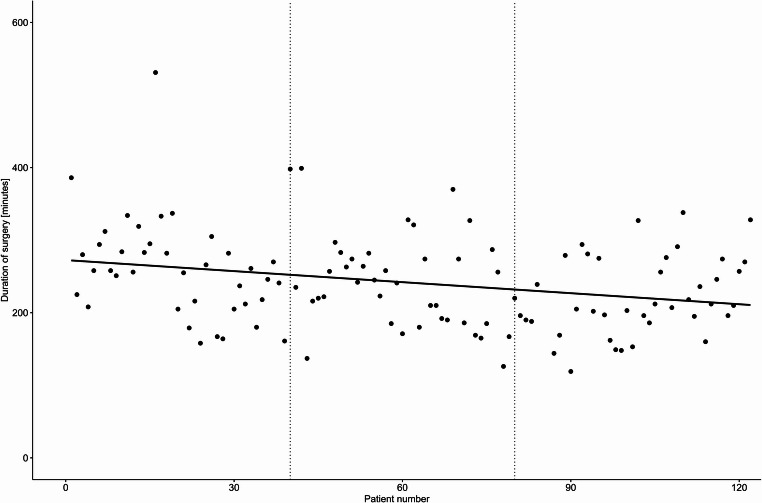
Duration of surgery in consecutive patients undergoing robotic distal pancreatectomy (*n* = 120)

Patients undergoing DP during the three defined time periods did not differ significantly in age, sex, BMI or ASA score (Table 2). The mean blood loss was highest in the two initial periods (230 ml vs. 247 ml vs. 133 ml, *P* = 0.192) however no patients required intra- or postoperative blood transfusion during any of the time periods. The rate of conversion from robotic to an open procedure was significantly reduced in the third time period (17.5% vs. 5.0% vs. 0%, *P* = 0.009). The mean duration of surgery decreased significantly from 263.8 min to 238.8 min and lastly 222.1 min per procedure (*P* = 0.003, Figure 1) and the rate of splenic preservation was highest in the last period (12.5% vs. 12.5% vs. 30%, *P* = 0.065). There was no difference in the median number of lymph nodes harvested in each period (12 vs. 14 vs. 12.5, *P* = 0.825).

**Table 2:** Pre- intra- and postoperative data on patients undergoing robotic distal pancreatectomy according to the experience of the surgical team BMI: body mass index. ASA: American society of anesthesiologists. ISGPS: international study group of pancreatic surgery. ∗: according to Müller et al. Ann Surg 2023;278:253–259

Variable	Level	Phase 1 (*n* = 40)	Phase 2 (*n* = 40)	Phase 3 (*n* = 40)	*P*	Benchmark value∗
Age [years]	mean (sd)	67.6 (12.8)	63.8 (12.5)	62.6 (15.3)	0.376	
Sex	Female	22 (55.0)	13 (32.5)	20 (50.0)		
	Male	18 (45.0)	27 (67.5)	20 (50.0)	0.106	
BMI [kg/m^2^]	mean (sd)	25.5 (4.3)	27.1 (5.1)	27.2 (4.6)	0.099	
ASA score	1	2 (5.0)	2 (5.0)	3 (7.5)	0.956	
	2	20 (50.0)	21 (52.5)	22 (55.0)		
	3	18 (45.0)	17 (42.5)	15 (37.5)		
Additional resection		1 (2.5)	4 (10.0)	0 (0.0)	0.066	
Splenic preservation		5 (12.5)	5 (12.5)	12 (30.0)	0.065	
Intraoperative blood loss [ml]	mean (sd)	230.2 (383.8)	247.2 (394.9)	132.9 (176.1)	0.192	
Conversion to open surgery		7 (17.5)	2 (5.0)	0 (0.0)	0.009	< 3%
Duration of surgery [minutes]	mean (sd)	263.8 (72.7)	238.8 (60.8)	222.1 (54.9)	0.003	< 300
Length of stay [days]	median (IQR)	6.5 [4]	6 [4.2]	6 [2.5]	0.156	
Postoperative pancreatic fistula	None	24 (60.0)	25 (62.5)	24 (60.0)	0.395	
	Biochemical	6 (15.0)	10 (25.0)	11 (27.5)		
	Grade B	10 (25.0)	5 (12.5)	5 (12.5)		< 32%
Clavien-Dindo complication grade						
	0	18 (45.0)	19 (47.5)	21 (52.5)	0.687	
	1	6 (15.0)	3 (7.5)	8 (20.0)		
	2	7 (17.5)	10 (25.0)	6 (15.0)		
	3a	7 (17.5)	4 (10.0)	4 (10.0)		
	3b	1 (2.5)	3 (7.5)	1 (2.5)		
	4	1 (2.5)	1 (2.5)	0 (0.0)		
Textbook outcome		25 (62.5)	28 (70.0)	33 (82.5)	0.134	
Pathology	Adenocarcinoma	12 (30.0)	10 (25.0)	11 (27.5)		
	IPMN	15 (37.5)	10 (25.0)	7 (17.5)		
	NET	8 (20.0)	9 (22.5)	9 (22.5)		
	Other	5 (12.5)	11 (27.5)	13 (32.5)	0.329	
Lymph nodes	median [IQR]	12 [11]	14 [12]	12.5 [10.5]	0.825	
Readmission		10 (25.0)	5 (12.5)	2 (5.0)	0.035	

Postoperative pancreatic fistula (POPF) grade B was found in 10 (25%) patients in the initial phase, 5 (12.5%) in the middle phase and 5 (12.5%) in the final phase (*P* = 0.162) and the median length of hospital stay was similar across periods (6.5 vs. 6 vs. 6, *P* = 0.156). The rate of patients with textbook outcome increased during the three phases of the study, however no significant differences between the three periods (62.5% vs. 70.0% vs. 82.5%, *P* = 0.134).

### CUSUM analysis

**Fig. 2:**
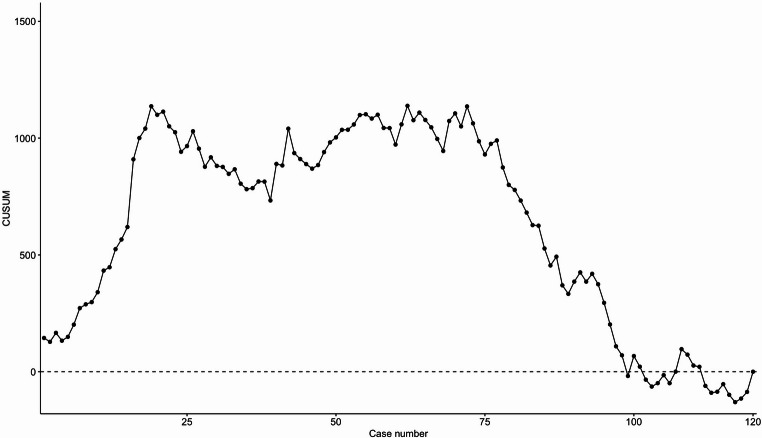
Cusum analysis of duration of surgery in robotic distal pancreatectomy

**Fig. 3:**
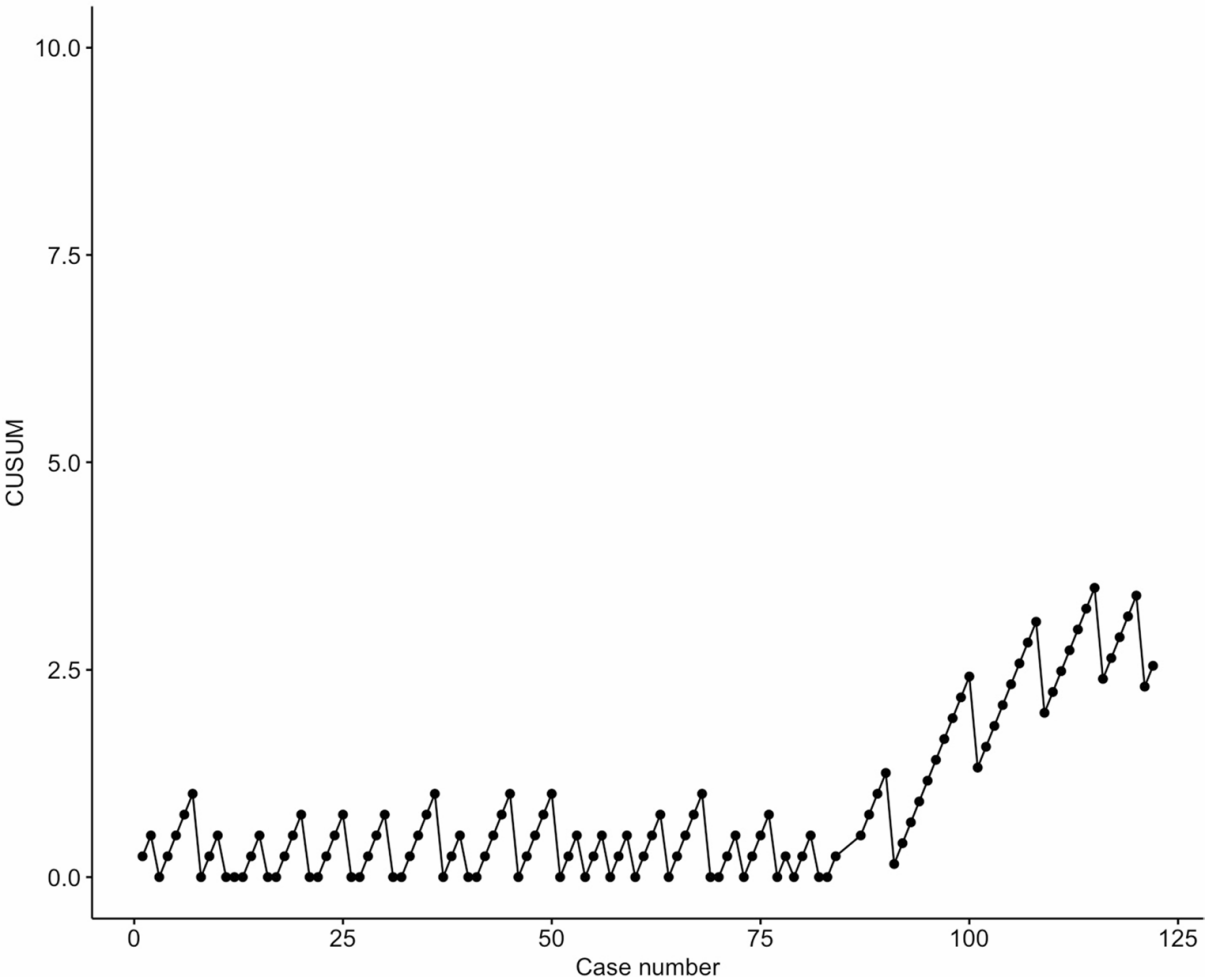
Cusum analysis of the Textbook Outcome after robotic distal pancreatectomy

The CUSUM analysis of duration of surgery and textbook outcome after RDP are shown in Figure 2 and 3, accordingly. The analysis of duration of surgery showed a peak at case number 19, and a significantly downwarding slope after case number 70. This indicated a stabilization of the learning curve after 19 procedures (the learning phase) and significant improvement after 70 procedures (transforming from the competency to the mastery phase). For the textbook outcome the CUSUM plot fluctuated around zero until case number 90, after which an upward slope on the graph was seen, indicating a stable process with expected rates of textbook outcomes until case number 90, where outcomes became better than expected.

#### PDAC

**Fig. 4:**
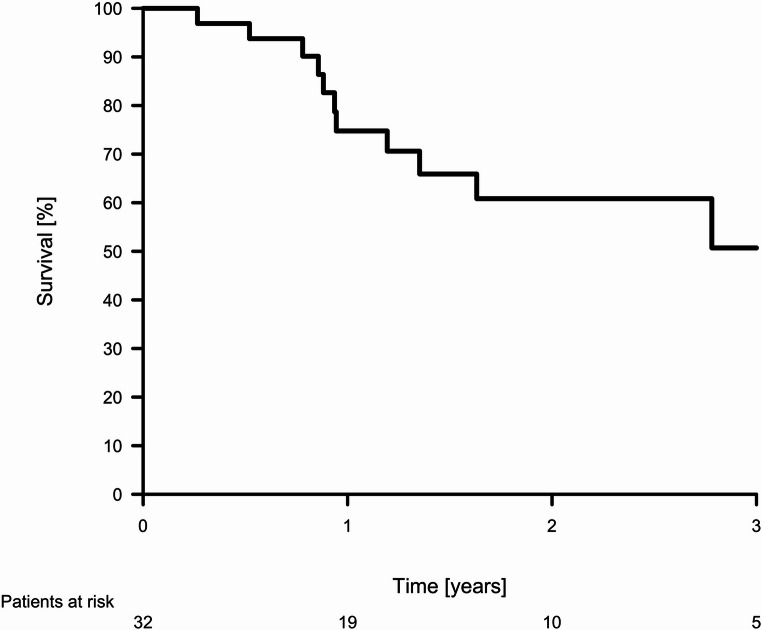
Survival of patients undergoing robotic distal pancreatectomy due to pancreatic ductal adenocarcinoma (*n* = 33)

During the study period 33 patients underwent RDP due to PDAC. In this subgroup the rate of conversion to open surgery was 12.1% and the mean duration of surgery was 275.8±75.1 min (Table 3). The total number of patients with no complications after RDP due to adenocarcinoma was 11 (33.3%). An R0 resection was achieved in 19 (57.6%) of the patients and no R2 resections were performed. Of patients with R1 resection, seven were on the anterior surface of the pancreas, three on the posterior and eight on both anterior and posterior. Lastly, one patient was graded as R1 due to relation to the stomach, which was also resected partly. Perineural and vascular invasion was found in 26 (78.8%) and 16 (48.5%) respectively. The median number of lymph nodes retrieved was 17 and positive lymph nodes were found in 18 (54.5%) patients. A total of 26 (78.8%) patients received adjuvant treatment, and the one- and three-year survival was 75.0% (CI 58.8–91.3) and 50.9 (CI 26.5–75.3), Figure 4.

**Table 3:** Pre- intra- and postoperative data on patients undergoing robotic distal pancreatectomy due to pancreatic adenocarcinoma

Variable	Level	Total (*n* = 33)
Age [years]	mean (sd)	69.8 (8.8)
Sex	Female	14 (42.4)
	Male	19 (57.6)
BMI [kg/m^2^]	mean (sd)	26.4 (4.6)
ASA score	II	15 (45.5)
	III	18 (54.5)
Additional resection		3 (9.1)
Intraoperative blood loss [ml]	mean (sd)	203.2 (251)
Conversion to open surgery		4 (12.1)
Duration of surgery [minutes]	mean (sd)	275.8 (75.1)
Length of stay [days]	median [range]	7 (5–52)
Postoperative pancreatic fistula	None	23 (69.7)
	Biochemical	8 (24.2)
	Grade B	2 (6.1)
Clavien-Dindo complication grade	0	11 (33.3)
	1	5 (15.2)
	2	11 (33.3)
	3a	3 (9.1)
	3b	3 (9.1)
	4	0 (0)
Textbook outcome		25 (75.8)
Tumorsize [mm]	mean (sd)	26.8 (12.8)
T stage	1	9 (27.3)
	2	19 (57.6)
	3	5 (15.2)
N stage	0	14 (42.4)
	1	14 (42.4)
	2	5 (15.2)
Lymph nodes	median [range]	17 [8–65]
Perineural invasion		26 (78.8)
Vascular invasion		16 (48.5)
Resection status	R1	20 (60.6)
Readmission		2 (6.1)
Adjuvant treatment		26 (78.8)
Recurrence during follow-up		7 (21.2)
Death during follow-up		12 (36.4)
Follow-up [days]	median [range]	447 [97 − 1,586]

BMI: Body mass index. ASA: American Society of Anesthesiologists. ISGPS: International Study Group of Pancreatic Surgery

## Discussion

In the current study we found the introduction of a robotic approach to DP to be safe with improvements in selected intra- and postoperative outcomes relative to the number of procedures performed.

We found that the duration of surgery decreased from the introduction and throughout the study period. This is in accordance with several other studies on the introduction of the robotic approach for DP [10, 11, 19–22]. In the current study the reduction in duration of surgery was probably related to improvement of several factors: Increasing experience with port placement and docking of the robotic system, increased robotic skill leading to reduced bleeding (although statistically insignificant) and faster dissection. The increasing rate of spleen preservation during the study period also impacted on the duration of surgery. The duration of surgery for patients with PDAC or main branch IPMN was prolonged to a varying degree due to the waiting for frozen sections.

It has previously been described that the plateau of the learning curve for minimally invasive DP is reached between 40 and 85 procedures depending on the outcome examined and was the basis for the division into three groups in the current study [11]. Other studies have examined the difference in learning curve comparing robotic and laparoscopic DP with equal number of procedures required for either approach [23]. To further examine the learning curve we performed the CUSUM analysis of the outcomes duration of surgery and textbook outcome. From this approach we found that the learning curve phase was surpassed after 19 procedures and mastery was obtained after 70 procedures in regard to duration of surgery. The rate of textbook outcomes was stable and acceptable until case number 90, where better-than-expected outcomes were obtained, again indicating mastery of the procedure.

For other robotic procedures the reported number of cases required to obtain proficiency varies from 25 to 80 depending on the type of procedure [24–26].

The incidence of postoperative grade B fistula (POPF B) was lowest in the final period of the study while the rate of splenic preservation was highest in this phase. This may represent proficient surgical skills reflecting the surgical team reaching the plateau of the learning curve [27]. Regarding other complications, the learning curve has been described to be around 50 procedures for duration of surgery, 70 procedures for blood loss and 40 procedures for conversion to open surgery [10, 20, 21, 28]. The results of the current study reflect these numbers well.

In the current study we described the transition from open to RDP, which is a genuine shift from open to a minimally invasive approach without changing the surgical strategy. Our institution is a high-volume HPB referral center and the surgeons performing the robotic procedures also perform transplant surgery, thus have vast experience with vessel dissection in open surgery. The laparoscopic approach to distal pancreatectomy has never been thoroughly introduced in our centre, considering the limitations of laparoscopic surgery. Robotic systems have a number of advantages over conventional laparoscopic surgery, including 7° of freedom when using Endowrist technology improved suturing capabilities, elimination of physiological tremor and superior surgeon ergonomics.　Besides, the advantage of the robotic system is that, compared to open surgery, it has the advantage that, although the field of view to be approached is different, the same surgical strategies can be reproduced as for the open approach with regard to the surgical technique. Nevertheless, there are downsides compared to laparoscopic approaches, such as increased costs and longer operative times. However, we decided to introduce a robotic system to DP without prior minimally invasive pancreas surgery experience in 2019 in the strong belief that, due to the advantages and strengths of the aforementioned robotic system, this technology will continue to exist and gradually develop. Furthermore, the caudal-dorsal approach to DP used in robotic surgery is different from the ventral approach used in open surgery, however, may have been easier to incorporate due to the advantages of the robotic system described above and the centre’s experience with transplant and donor surgery. As this was a retrospective cohort study of consecutive patients, naturally, the selection of patients was biased. With increasing experience the indication for RDP widened, however without protocolled changes. This is difficult to objectively analyze, however, a cautious approach to patient selection allowed for steady increase in the surgical complexity of the procedures.

Several studies have described the outcomes of a change from open to laparoscopic approach, reporting reduced blood loss and postoperative length of stay after laparoscopic DP [29]. In general, minimally invasive compared to open procedures lead to a reduced length of stay, related to the less surgical trauma in minimally invasive surgery. In the current study, the length of stay was not highly different from what has been reported after open DP, which may be explained by several factors. First, the surgical drain placed intraoperatively remains at least until postoperative day four, to examine the drain fluid for potential signs of pancreatic leakage. Second, the surgical trauma to the abdominal wall is probably less relevant with regards to discharge after the first four days, rather what keeps the patient in the hospital after DP is recovery of sufficient oral intake or minor complications reflected in the complication rates of the current study. We chose to implement a robotic approach to DP since this mimic the open approach well, whereas laparoscopic approach has more technical differences. Although not sufficiently described in the literature currently, we strongly believe that robot-assisted DP will eventually lead to superior outcomes compared to open and laparoscopic approach if patient selection is done adequately. In our experience, case observation at established high-volume robotic centers followed by proctoring from leaders in the robotic surgical community allows for the optimal implementation of a robotic pancreatic surgery program.

The outcomes presented in the current study are comparable to what has previously been reported from high-volume pancreatic centers, in terms of duration of surgery, complication rate (Clavien-Dindo ≥ 3) and incidence of postoperative pancreatic fistula [30, 31]. We found that the rate of spleen preservation increased with the number of cases performed, possibly reflecting increased skill proficiency. A similar pattern has been described in previous publications on the learning curve of RDP, however, it may be difficult to further interpret due to the pathologic heterogenicity of the included patients [31]. Overall, studies point towards a higher incidence of spleen preservation in robotic compared to laparoscopic DP at rates comparable to what was found in our study [32, 33].

A recent study evaluated benchmark cutoff values for RDP based on data from sixteen high-volume centers, including some patients from this cohort [27]. The overall rate of conversion to open surgery and grade B as well as C leakage was above the proposed benchmark cutoffs in the first two time periods, while the benchmark was reached in the third period. For the duration of surgery and major complications the results for all three time periods in the current study were below the proposed benchmark values. Thus, it seems that the benchmark values are difficult to reach during the learning curve of RDP, while introduction of this approach was safe with regards to major adverse events. This was further supported by the steady rate of textbook outcomes across the three time periods of the current study, in accordance with a recent large nationwide cohort study from the Dutch pancreatic cancer audit [34].

For patients with PDAC the benchmark number of resected lymph nodes was obtained, however benchmark for R0 resection rate as well as one- and three-year survival was not reached, probably due to the higher incidence of T2 and T3 tumors, perineural and vascular invasion and positive lymph nodes in the current cohort. We do not believe that the relatively low rate of R0 resections was directly associated with the robotic approach, rather a consequence of a high rate of R1 resections due to intimate relationship with the anterior or posterior pancreatic surface. Further, differences in the definition of resection margins in pancreatic resections may explain the variation across the current and other studies [15].

The current study has some limitations. As we analyzed the learning curve of the entire team of three surgeons, there may be an inherent bias as the three may climb the learning curve at different paces. Further, there is a high risk of selection bias as patients selected for RDP in the initial phase of the study cohort were intentionally considered less technically challenging, to facilitate favorable outcomes of a new technique. Lastly, the relatively low number of patients with PDAC limits the interpretation of the outcomes of this specific subgroup due to a high risk of type-II error. We did not examine the duration of surgery with respect to each single surgeon for two reasons. First, the aim of the study was to report the outcomes of RDP introduction for a dedicated team, and as such it was anticipated that the experience of the team members would increase gradually regardless of who was the console surgeon and assistant. Secondly, the console surgeon was changed in some cases thus leaving it impossible to get valid data on the duration of surgery on a single surgeon level.

In conclusion we found that RDP can be safely introduced without prior experience with laparoscopic DP, surpassing the learning curve after 19 procedures and reaching benchmark outcome values after 60 procedures.

## Data Availability

No datasets were generated or analysed during the current study.
